# Structural analysis of the SAM domain of the Arabidopsis mitochondrial tRNA import receptor

**DOI:** 10.1016/j.jbc.2024.107258

**Published:** 2024-04-04

**Authors:** Bence Olasz, Luke Smithers, Genevieve L. Evans, Anandhi Anandan, Monika W. Murcha, Alice Vrielink

**Affiliations:** School of Molecular Sciences, The University of Western Australia, Perth, Western Australia, Australia

**Keywords:** mitochondria, tRNA import, tric1, SAM domain, oligomerization, superhelix, protein structure, PRAT domain

## Abstract

Mitochondria are membrane-bound organelles of endosymbiotic origin with limited protein-coding capacity. The import of nuclear-encoded proteins and nucleic acids is required and essential for maintaining organelle mass, number, and activity. As plant mitochondria do not encode all the necessary tRNA types required, the import of cytosolic tRNA is vital for organelle maintenance. Recently, two mitochondrial outer membrane proteins, named Tric1 and Tric2, for tRNA import component, were shown to be involved in the import of cytosolic tRNA. Tric1/2 binds tRNA^ala^*via* conserved residues in the C-terminal Sterile Alpha Motif (SAM) domain. Here we report the X-ray crystal structure of the Tric1 SAM domain. We identified the ability of the SAM domain to form a helical superstructure with six monomers per helical turn and key amino acid residues responsible for its formation. We determined that the oligomerization of the Tric1 SAM domain may play a role in protein function whereby mutation of Gly241 introducing a larger side chain at this position disrupted the oligomer and resulted in the loss of RNA binding capability. Furthermore, complementation of *Arabidopsis thaliana* Tric1/2 knockout lines with a mutated Tric1 failed to restore the defective plant phenotype. AlphaFold2 structure prediction of both the SAM domain and Tric1 support a cyclic pentameric or hexameric structure. In the case of a hexameric structure, a pore of sufficient dimensions to transfer tRNA across the mitochondrial membrane is observed. Our results highlight the importance of oligomerization of Tric1 for protein function.

Mitochondria are essential organelles in many eukaryotic cells. The mitochondria arose over 1 billion years ago in an event where an archaebacterium and an eubacterium formed a symbiotic relationship (1). Due to the endosymbiotic origin, mitochondria maintain an active genome, encoding mitochondrial proteins and RNA products required for mitochondrial function. The number of active protein-coding mRNA, tRNA, and rRNA-coding genes varies significantly between eukaryotes (2). While much of the mitochondrial protein content is nuclear-encoded, synthesized in the cytosol as preproteins, and imported into the mitochondria *via* the TOM and TIM complexes, some of the mitochondrial protein content is encoded by the organelle genome. The mitochondrial genome encodes a number (varying between organisms) of genes to produce different tRNA species; however, some tRNA, required for proper organelle function, is also imported from the cytosol.

In *Arabidopsis thaliana* (*Arabidopsis*), a plant-specific mitochondrial tRNA import component has been identified (3). Named Tric1 and Tric2 (tRNA import component 1 and tRNA import component 2), these almost identical proteins belong to the preprotein and amino acid transporters (PRAT) family of proteins but are distinct from other PRATs in that they also contain a C-terminal SAM (sterile-alpha-motif) domain (4).

The PRAT protein family is a large, conserved family of membrane proteins responsible for the transport of proteins and amino acids into both mitochondria and plastids (4, 5, 6, 7, 8, 9). In *Arabidopsis*, 10 of the 16 members of the PRAT family are found in the mitochondrial inner membrane and include the protein import transporters Tim17, Tim22, and Tim23 (6). Another three, including OEP16, are located in the chloroplast outer envelope and have been shown to be involved in amino acid transport (6). Tric1 and Tric2, have been shown to be dual-targeted to both the mitochondria and chloroplast and to be involved in tRNA import, with the SAM domain shown to bind tRNA directly (3).

SAM domains, first characterized in yeast and *Drosophila melanogaster* (10) are composed of ∼65 to 70 amino acids and participate in diverse developmental processes including sexual differentiation in yeast and spatial regulation during embryonic development in *D. melanogaster* (11). Since the discovery of this domain, it has been identified in numerous diverse proteins in a wide range of eukaryotes (12, 13, 14), including plants (15, 16), and even in some prokaryotes (17).

The SAM domain has originally been described as a protein-binding domain, involved in protein–protein interactions. They have been observed in head-to-tail homo-SAM interactions (18, 19, 20) or hetero-SAM interactions (21, 22) forming linear oligomeric structures usually consisting of six monomers per turn. Other stoichiometries have also been observed, for example, in LEAFY (15) or in PHC3 SAM domains form five monomers per helical turn (14). SAM domains have also been observed in heterotypic protein interactions such as shown in Ets2, a human transcription factor, binding Cdk10 (cyclin-dependent kinase 10) with the interaction implicated in transactivation (23) or BAR (bifunctional apoptosis regulator) interacting with Bcl-2 (B-cell lymphoma-2) and Bcl-X_L_ (B-cell lymphoma-extra large) where they play a role in regulating apoptosis (24). Furthermore, a mammalian scaffolding protein, CASKIN1, and its homolog, CASKIN2, contain two SAM domains. In CASKIN2, the tandem SAM domain forms an oligomeric structure, where the minimal repeating unit is a dimer, in contrast to the previously observed monomeric repeating unit, adding further variety to the mode of oligomerization of SAM domains (25). Interestingly, the presence of the SAM domain in EphA2, a human receptor tyrosine kinase, inhibits ligand-independent clustering of the receptor and kinase activity, thus affecting the phosphorylation of EphA2 target proteins (26, 27).

In addition to protein–protein interactions, SAM domains have been observed to interact with RNA. The first SAM domain-containing protein shown to bind RNA was Smaug from *D. melanogaster* (28, 29). The structure of Vts1, a yeast RNA binding protein and homolog of Smaug, has been solved by NMR spectroscopy (30), describing the RNA binding surface of the SAM domain and identifying key residues required for RNA interaction. In *Arabidopsis* Tric1 and Tric2 SAM domains have been shown to bind tRNA^ala^ *via* key lysine residues at positions 205 and 210 and are required for tRNA import into the mitochondria *in vivo* (3).

Here we describe the crystal structure of the SAM domain from Tric1. We show it has the ability to oligomerize to form a helical superstructure with six SAM monomers per helical pitch and identify key residues required for this oligomerization. *In vitro* RNA binding assays reveal these residues are also required for RNA binding and complementation of the double Tric1 and Tric2 knockout mutant line (*tric1:tric2*). Finally, modeling with AlphaFold2 of the full-length Tric1 protein supports the hexameric form hinted at in the crystal structure.

## Results

### The crystal structure of WT Tric1 SAM domain

**Figure 1:**
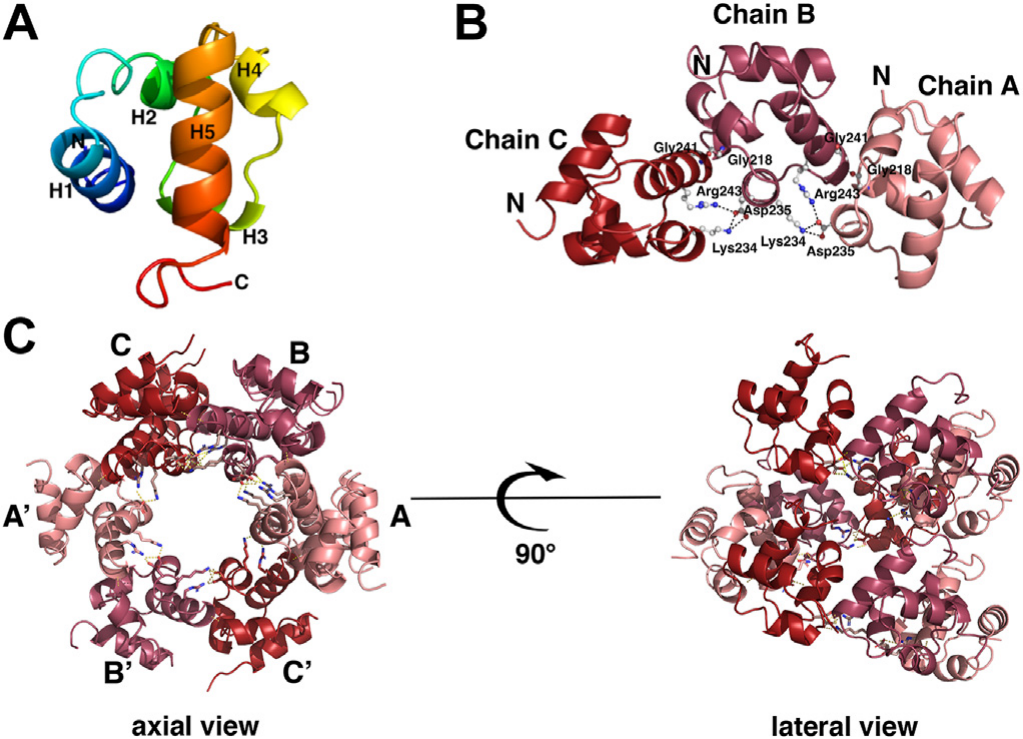
**The structure of the *Arabidopsis thaliana* Tric1 SAM domain.***A*, a ribbon representation of the monomer showing the secondary structure elements. The protein is colored from *blue* for the N-terminus to *red* for the C-terminus with the five alpha helices labeled. *B*, the intermolecular interactions between the monomers of the asymmetric unit in the WT structure. Each monomer is shown in cartoon representation and colored different shades of *red*. The monomers are labeled as Chain (*A*–*C*). The residues involved in the inter-subunit interactions are shown in *stick* representation with the hydrogen bonds displayed as *dashed lines*. *C*, the superstructure formed by the WT Tric1 SAM domain corresponds to three asymmetric units in the crystal lattice shown from an axial and a lateral view. The monomers in one asymmetric unit are labeled as (*A*–*C*), and the corresponding monomers in the second asymmetric unit are labeled as (*A*’–*C*’). The third asymmetric unit monomers lie directly below those of the first asymmetric unit.

The crystal structures of the Tric1 SAM domain (residues 191–261) as well as two variants (Asp235Ala and Gly241Glu) have been determined. The wildtype (WT) SAM domain structure was determined by MAD phasing from a selenomethionine mutant; the protein chain has three methionine residues including the first methionine in the N terminal Histidine tag however only the methionine residues from the SAM domain (Met202 and Met 245) were used for phasing. The final structural model was refined to 1.48 Å resolution. The asymmetric unit of the crystal for the WT structure contains three monomers with identical contacts between the three protein chains. The protein adopts a characteristic fold consisting of a 5-alpha-helical bundle (Fig. 1*A*). The overall globular structure consists of four short helices (H1-4) and one long helix (H5) at the C-terminal end of the protein. Comparison to other SAM domains (14, 15, 28, 30) indicates that the overall fold of the protein and secondary structure elements are highly conserved and that the Tric1 SAM domain has slightly more loop regions and shorter helices (Fig. S1*A*). The observed electron density for the entire protein chain is well defined, with the exception of three positively charged residues at the C-terminus (Lys259, Arg260, Lys261). This structural conservation between SAM domains in different proteins is common despite low sequence conservation (Fig. S1*B*). Low sequence identity is observed between SAM domains involved in RNA binding (Tric1, Vts1, and Smaug) and those not involved in RNA binding (LFY and PHC3) (Fig. S1*B*).

The oligomeric structure within the asymmetric unit shows a number of side chains that are involved in hydrogen bond and salt bridge contacts between the subunits (Fig. 1*B*). Specifically, the side chain of Asp235 of one subunit makes hydrogen bond contacts to the side chains of Lys234 and Arg243 of the adjacent subunit. Additionally, the main chain carbonyl atom of Gly218 of one subunit makes a hydrogen bond contact with the main chain nitrogen atom of Gly241 of the adjacent subunit (Fig. 1*B*). Further analysis of the crystal lattice revealed a helical superstructure, similar to other SAM domains (14, 19, 20, 31, 32). This superhelix consists of six monomers per turn with a pitch of 28 Å (Fig. 1*C*). An electrostatic surface of the superhelix reveals a positively charged surface along one face of the six-subunit structure near the amino end of the SAM domain and a negatively charged surface along the opposite face of the structure near the carboxyl end of the SAM domain (Fig. S2*A*). This charge complementarity likely contributes towards the lattice packing. It is noteworthy that residues prior to the amino end of the SAM domain in the full-length protein may affect the electrostatics of this surface. Intermolecular subunit interactions are more extensive (397 Å^2^ buried surface), as calculated using the PDBePISA tool: Proteins, Interfaces, Structures and Assemblies (33), between adjacent molecules laterally than between molecules along the pitch of the superhelix (251 Å^2^ buried surface). Besides the lateral interactions, intermolecular hydrogen bond interactions between the side chain nitrogen atom of Lys217 of one monomer and the main chain oxygen atoms of Ile250, Asp253 and Ile256 at the C-terminus of the domain on a neighboring symmetry related monomer parallel to the helical axis further strengthen the superstructure formed in the crystal lattice (Fig. S2*B*).

**Figure 2:**
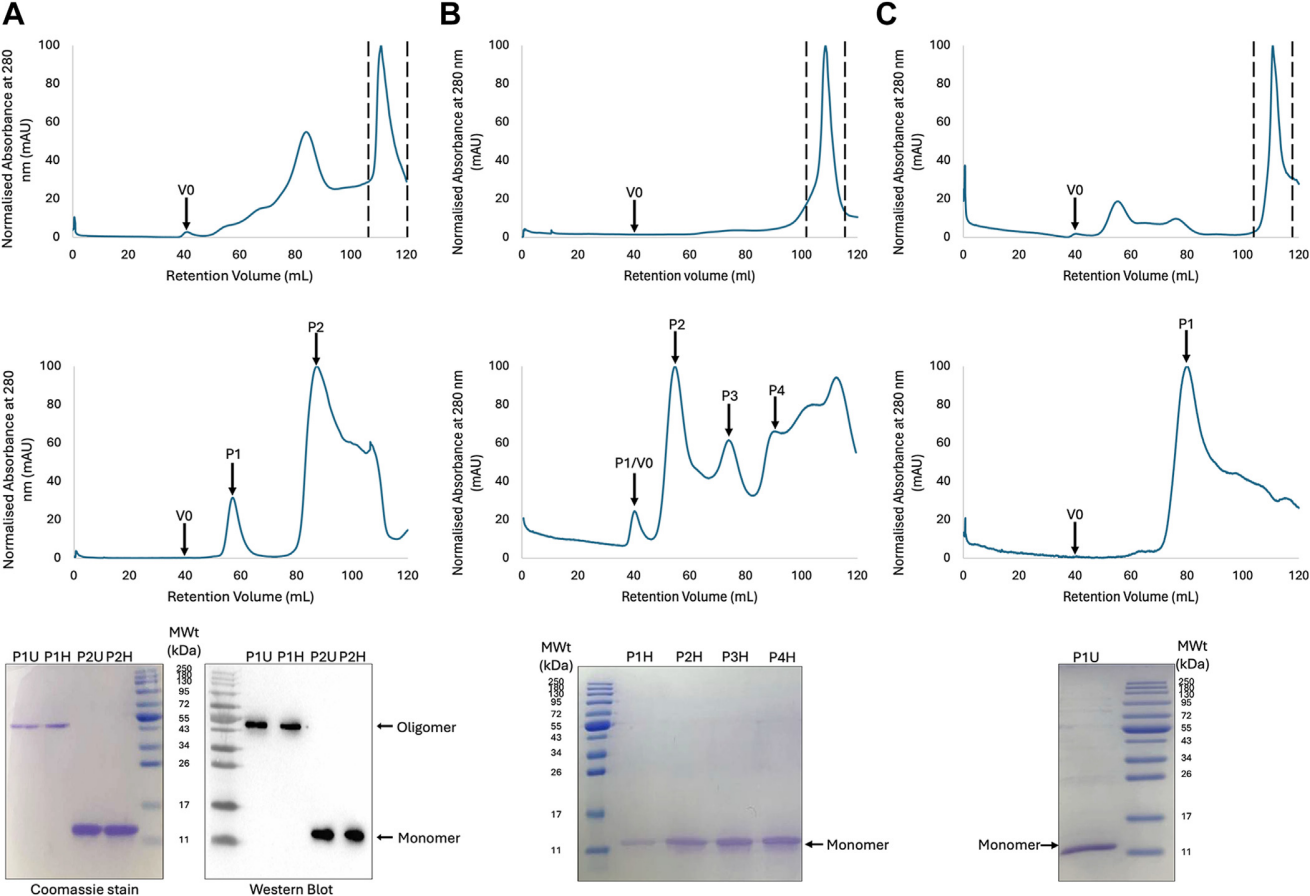
**Gel filtration and SDS-PAGE analysis of SAM domain.** All gel filtration chromatograms show the void volume (V0) and are plotted with normalized absorbance, where the highest absorbance value is set to 100. All SDS-PAGE and Western blots refer to the peaks indicated in the panel directly above them, where U = unheated and H = heated (95 °C for 10 min). *A*, WT SAM domain. *Top*: S300 gel filtration purification. Two *vertical lines* indicate the samples taken and loaded on the S75. *Middle*: S75 gel filtration chromatogram. Two peaks are indicated (P1, P2). *Bottom*: SDS-PAGE and Western blot analysis of P1 and P2 from S75 gel filtration. *B*, SAM Asp235Ala mutant. *Top*: S300 gel filtration purification (as for *A* top). *Middle*: S75 gel filtration chromatogram. 4 peaks (P1-P4) are indicated. *Bottom*: Analysis by SDS-PAGE of chromatogram peaks. *C*, SAM Gly241Glu mutant. *Top*: S300 gel filtration purification (as for *A* top). *Middle*: S75 gel filtration chromatogram. 1 peak (P1) is indicated. *Bottom*: SDS-PAGE analysis of P1.

In order to assess whether the oligomeric structure observed in the crystal structure is also present in the solution, pure WT SAM domain was eluted from a Superdex75 gel filtration column, and the peaks were analyzed by Western Blot (Fig. 2*A*). Pure SAM domain eluted from the column as two main species, an oligomer at a retention volume of ∼58 ml, and a further peak at a retention volume of ∼90 ml, representing the monomeric SAM domain (Fig. 2*A*). Samples from each of the peaks were heat treated or left unheated prior to loading on an SDS gel followed by Western Blot analysis using a monoclonal anti-Poly histidine-peroxidase antibody to identify specifically the histidine-tagged version of the SAM domain. Interestingly, both the heated and unheated oligomeric samples appeared on the blot at approximately 50 kDa, a higher molecular weight than would be expected for the monomer (∼11 kDa). This suggests that WT SAM domain forms a highly thermally stable oligomeric form in solution (Fig. 2*A*). It should be noted that a folded, and therefore more compact protein, is expected to electrophorese faster on the gel and appear as a smaller sized species than a completely unfolded protein (Fig. 2*A*). This appears to be the case for the WT SAM domain oligomer, as we assume the protein remains folded in order to retain its discrete oligomeric state. This result provides evidence that the SAM domain forms a thermally stable oligomeric species, likely larger than 50 kDa. To the best of our knowledge, this is the first example of such a thermally stable SAM oligomeric complex. It should be noted that there are no cysteines in the protein sequence to contribute to inter or intramolecular covalent bonds.

### An intermolecular interaction involving Gly241 and Gly218 is required for the formation of the Tric1 SAM domain helical superstructure

To determine if the homo-oligomerization ability of the Tric1 SAM domain is required for protein function, mutational studies were undertaken. Based on the structural analysis of Tric1 SAM domain and the interactions between monomers in the helical superstructure, several mutants were designed. The Aps235Ala mutant would eliminate the salt bridge interaction between the carboxylate group of Asp235 and the side chains of Lys234 and Arg243 of a neighboring monomer. Additionally, the Gly241Glu mutation is hypothesized to push two neighboring monomers away from one another due to the extended glutamate side chain as well as introducing a negative charge.

**Figure 3:**
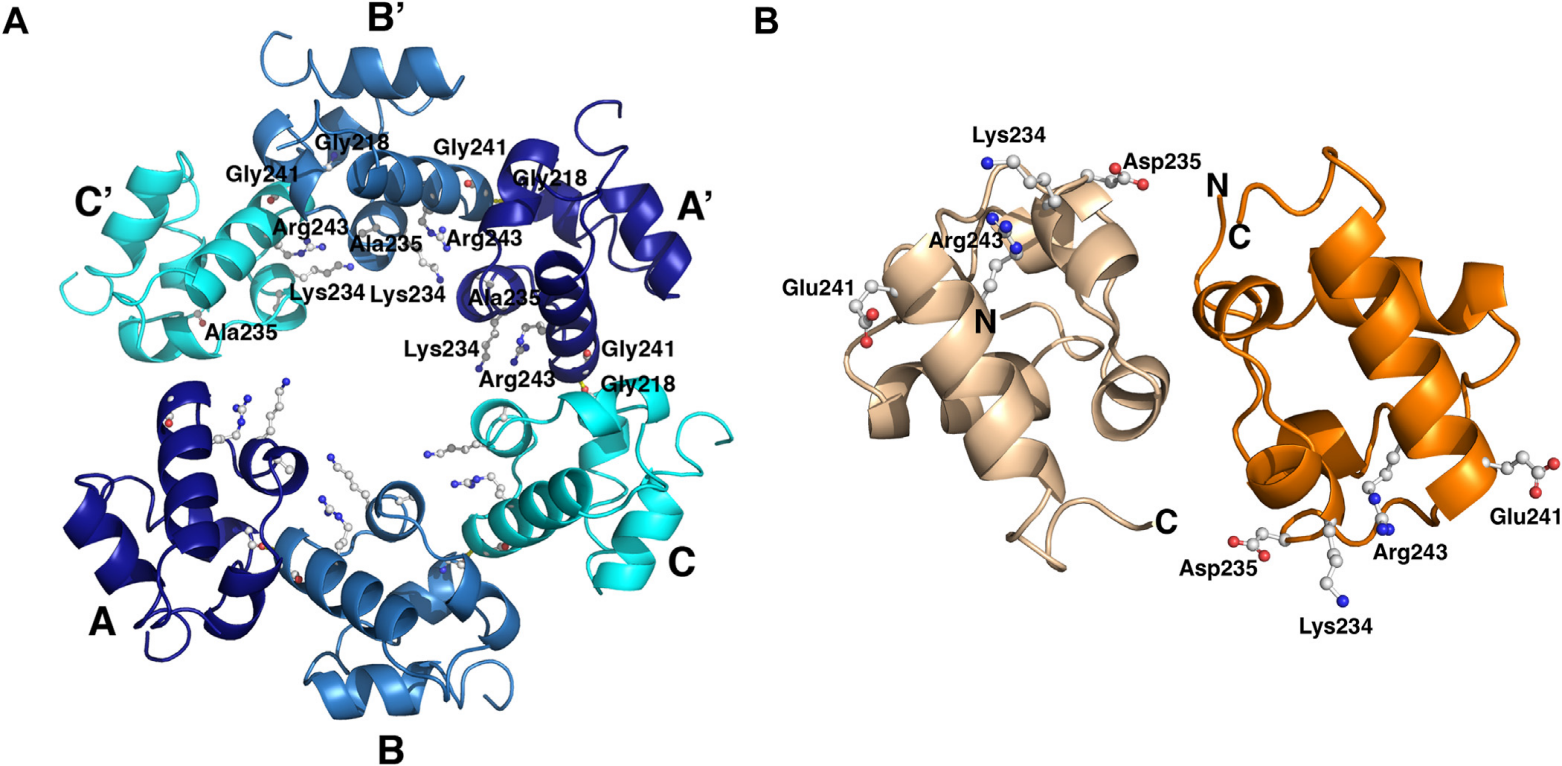
**Structures of the SAM domain variant proteins.***A*, the Asp235Ala variant structure shows two asymmetric units corresponding to six monomers. The monomers in one asymmetric unit are labeled as (*A*–*C*) and those in the second asymmetric unit are labeled as (*A*’–*C*’). The monomers are shown as a ribbon representation in different shades of *blue*. *B*, the non-crystallographic symmetry dimer in the asymmetric unit of the Gly241Glu structure. The N- and C-termini for each monomer in the Gly241Glu structure are labelled. Each monomer is shown in a different shade of *orange*. Individual amino acid side chains are shown in *ball* and *stick* representation.

The crystal structure of the Tric1 SAM Asp235Ala mutant was determined and refined to 2.07 Å resolution revealing an identical fold and lattice packing to that of the wild-type (WT) protein (Fig. S4, *A* and *B*). As expected, the salt bridge interaction between position 235 and Lys234 and Arg243 is absent; hydrogen bond interactions are still present between the main chain oxygen of Gly218 of monomer A and the main chain nitrogen of Gly241 of monomer B (Fig. 3*A*). Additionally, the mutant adopted an identical super helical structure to that observed for the Tric1 SAM domain (Fig. S3, *A* and *B*). Each of the monomers in the asymmetric units of the crystal structure of the Asp235Ala mutant superimposed closed onto the monomers of the WT structure (Fig. S3*D*) showing that the helical superstructure was identical for the two structures.

Crystals of the Gly241Glu mutant were obtained but from different crystallization conditions, after trials using the crystallization conditions identified for WT and Asp235Ala failed to produce diffraction quality crystals, suggesting a different packing to the WT SAM domain and to the Asp235Ala mutant. Furthermore, despite the identical space group for all three structures, the unit cell dimensions of the Gly241Glu mutant crystal structure differed from those of the WT Tric1 SAM domain and the Asp235Ala mutant (Table 1). The structure was determined and refined to 1.89 Å resolution. While the overall fold of the monomer does not differ from that of the WT protein or the Asp235Ala mutant, the number of molecules in the asymmetric unit and the overall crystal packing did differ significantly. Two molecules are present in the asymmetric unit and the polar intermolecular contacts between the two monomers were notably absent (Fig. 3*B*). Furthermore, the crystal lattice no longer showed a helical superstructure (Fig. S3*C*). The arrangement of the two molecules in the asymmetric unit of the Gly241Glu mutant could not be superimposed onto two molecules of the trimer in the WT structure indicating a different protein-protein interface between the WT and mutant (Fig. S3*E*). Specifically, the close approach of the monomers for the WT protein due to the presence of a glycine at position 241, leading to a hydrogen bond between the main chain nitrogen of Gly241 and the main chain oxygen of Gly218 on the neighboring molecule is not possible when the glycine is replaced with a longer glutamate side chain (in the Gly241Glu mutant). Thus, the introduction of a longer side chain at position 241 in the amino acid sequence was sufficient to eliminate the subunit arrangement of the structure in the crystal lattice.

**Table 1:** Crystallographic data collection and refinement statistics

Protein	Se-Met				WT	Asp235Ala	Gly241Glu
	Inflection point	Peak	Low remote	High remote			
Beamline	AS MX1	AS MX1	AS MX1	AS MX1	AS MX1	AS MX1	AS MX2
Data collection statistics							
Space group	*P*2_1_2_1_2_1_	*P*2_1_2_1_2_1_	*P*2_1_2_1_2_1_	*P*2_1_2_1_2_1_	*P*2_1_2_1_2_1_	*P*2_1_2_1_2_1_	*P*2_1_2_1_2_1_
Unit cell dimensions (Å)					*a* = 28.6 *b* = 92.1 *c* = 93.3	*a* = 28.7 *b* = 92.6 *c* = 94.1	*a* = 46.6 *b* = 58.5 *c* = 61.9
Resolution (Å)^a^					1.48 (1.53–1.48)	2.07 (2.14–2.07)	1.89 (1.96–1.89)
Total reflections	99,311	99,689	100,670	16,1549	599,785 (52,161)	220,700 (20,864)	183,488 (18,001)
Unique reflections	20,597	20,608	20,709	35,642	42,131 (4135)	15,930 (1505)	14,037 (1355)
Completeness (%)					99.9 (99.9)	97.3 (97.7)	99.5 (96.1)
*R*_*merge*_					0.05 (1.20)	0.14 (0.72)	0.09 (1.50)
*R*_*pim*_					0.01 (0.34)	0.03 (0.019)	0.02 (0.42)
Average I/σ					36.3 (2.1)	19.5 (4.0)	18.9 (2.4)
Multiplicity					14.2 (12.6)	13.9 (13.9)	13.1 (13.1)
CC_½_					1 (0.75)	1.0 (0.91)	1.0 (0.78)
Refinement statistics							
*R*_*work*_					0.18 (0.25)	0.25 (0.28)	0.20 (0.29)
*R*_*free*_					0.20 (0.28)	0.27 (0.34)	0.24 (0.31)
# Protein residues					198	193	125
# Solvent molecules					180	226	81
RMS (bonds) (Å)					0.012	0.005	0.004
RMS (angles) (^o^)					1.2	0.8	0.8
Ramachandran favoured (%)					99.5	98.9	97.5
Ramachandran outliers (%)					0	0	0
Average B-factors (Å^2^)					29.3	26.1	41.78

a Values in parentheses are for the highest resolution shell.

Gel filtration experiments were carried out with the Asp235Ala and the Gly241Glu mutants, identical to those carried out on the WT (Fig. 2, *B* and *C*). The Asp235Ala mutant eluted from the S75 as a series of different sized oligomers and monomeric protein. None of these displayed the same thermal stability as the WT protein, suggesting this mutation has severely crippled the ability of the protein to form a stable, discrete oligomeric species in solution. The Gly241Glu protein eluted entirely in the monomeric form, supporting our observations from the crystal structure that this mutation disrupts the ability of the protein to oligomerize entirely.

### Asp235 and Gly241 are important for SAM domain-mediated RNA binding

**Figure 4:**
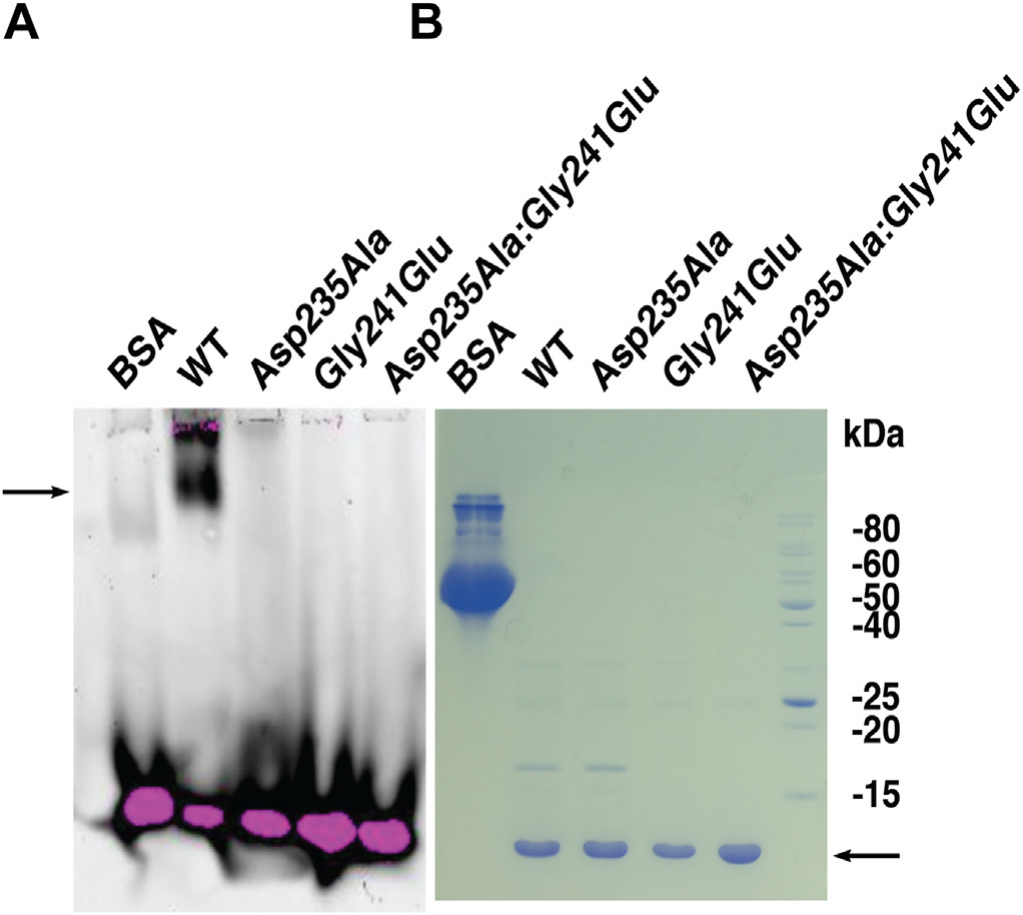
**RNA Electrophoretic mobility shift assays of Tric1 SAM domain.***A*, WT Tric1 SAM domain and the variants Asp235Ala, Gly241Glu, and Asp235Ala:Gly241Glu were electrophoresed with a fluorescein-labeled T-arm of tRNA^ala^. The *arrow* indicates the position of the WT Tric1 SAM domain shifted band. *B*, all the protein samples used in the electrophoretic mobility shift assay were resolved using 15% SDS-PAGE and Coomassie stained. The *arrow* indicates the position of the purified Tric1 SAM domain protein and variants.

It was previously shown that the recombinant Tric1 SAM domain (residues 191–261) binds the T-arm of tRNA^ala^ *via* conserved lysine residues at positions 205 and 210 (numbered as Lys15 and Lys20 of the SAM domain alone) (3). To determine if the oligomerization ability of Tric1 SAM domain was required for this binding, the RNA binding ability of Tric1 SAM Asp235Ala and Gly241Glu variants was tested. Electrophoretic mobility shift assays (EMSA) using fluorescein-labeled tRNA^ala^ oligonucleotides was carried out (Fig. 4). Incubation of Tric 1 SAM domain with tRNA^ala^ resulted in a shifted band indicating RNA binding, as previously observed (3). No mobility shift bands were observed for the Tric1 SAM variants Asp235Ala, Gly241Glu, or the double variant Asp235Ala:Gly241Glu suggesting the abolishment of the tRNA-binding capacity (Fig. 4*A*). Coomassie staining confirms purity and equal concentration of recombinant protein used in the assay (Fig. 4*B*)

### Mutation of Asp235 and Gly241 abolishes Tric1 protein function *in planta*

**Figure 5:**
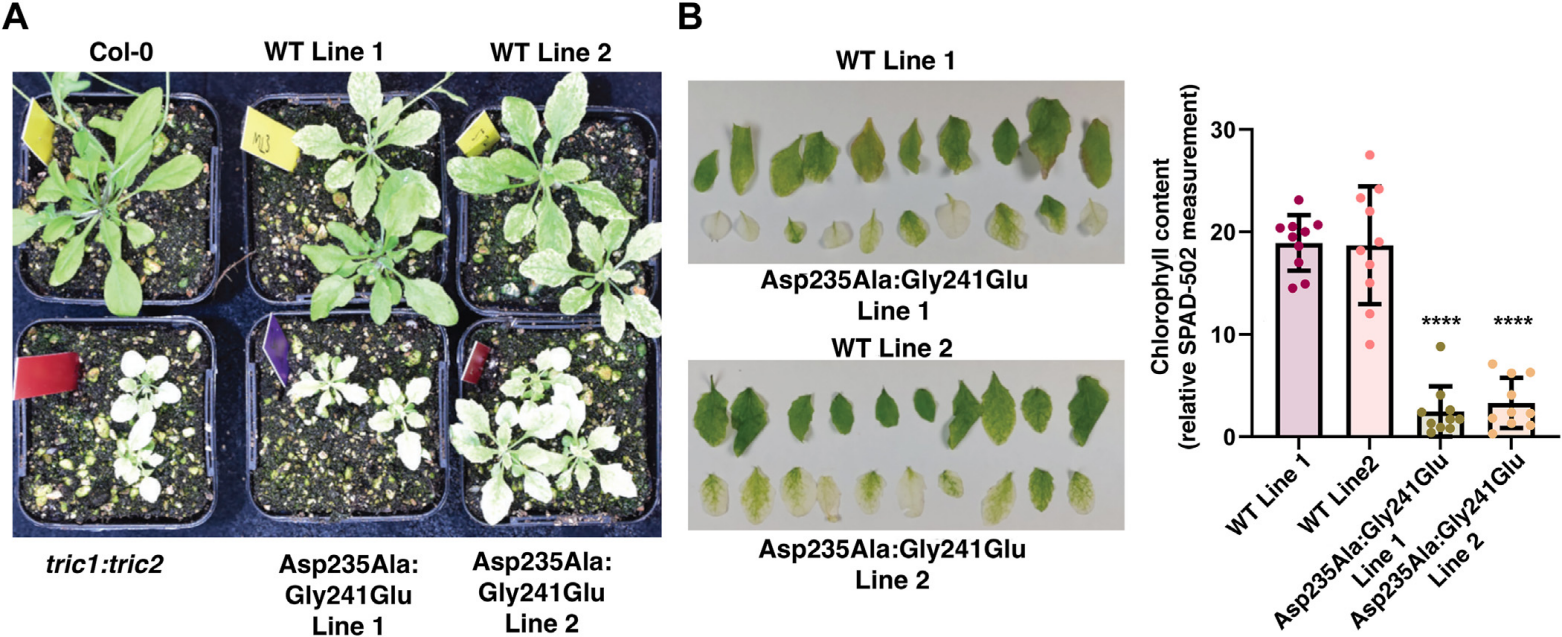
**The effect of key amino acid mutations on Tric1 function *in vivo*.***A*, complementation of the *Arabidopsis thaliana tric1:tric2* mutant with full-length Tric1 (WT Lines 1 & 2) and the Asp235Ala:Gly241Glu variant of Tric1 (Asp235Ala:Gly241Glu Lines 1 & 2). *B*, chlorophyll content was compared for all complemented plant lines using a handheld SPAD-502 chlorophyll meter. Each bar shows the relative Special Products Analysis Division (SPAD) units from five technical replicates across one leaf from 10 individual plants. Significant differences are indicated by ∗∗∗∗ (±sd, *p* < 0.0001, one way ANOVA from the Brown-Forsythe and Welsch tests, n = 10). Col-0 = *Arabidopsis thaliana* ecotype Columbia.

Biochemical assays suggest that Asp235 is required for tRNA binding, and Gly241 is required for oligomer formation and tRNA binding (Fig. 4). To determine if these residues are essential for Tric1 function *in planta*, complementation assays were carried out on the *tric1:tric2* knockout line using the full-length WT Tric1, and Asp235Ala:Gly241Glu variants. The *tric1:tric2* knockout exhibits a distinct small, pale, and developmentally delayed phenotype, which could be restored by complementation with Tric1. This complementation assay was repeated and shown to again restore the defective phenotype (Fig. 5*A*) (3). Independent complemented lines: WT 1 and WT 2, exhibited larger plants with significantly increased chlorophyll content (Fig. 5, *A* and *B*). Complementation of *tric1:tric2* knockout with the double mutant variant Asp235Ala:Gly241Glu failed to restore the defective growth and/or the chlorotic phenotype (Fig. 5, *A* and *B*). Two independent complementation lines: Asp235Ala:Gly241Glu exhibited the small developmentally delayed growth phenotype with significantly decreased chlorophyll content compared to WT complementation lines (Fig. 5*B*). These results suggest that Asp235, Gly241, or both are required for functional complementation *in planta via* tRNA binding, oligomerization ability, or both.

### Structure predictions of the SAM domain and Tric1 support an oligomeric arrangement for tRNA import

It is unlikely that the SAM domain will adopt a super helical structure as observed in the crystal structure in the context of the full-length structure which includes the PRAT domain, anchoring the protein in the mitochondrial membrane. One might expect, however, that a closed cyclic assembly could occur in the presence of the transmembrane domain.

To assess the possible oligomeric arrangement of the protein, structure predictions were performed. The artificial intelligence program, AlphaFold, developed by Deep Mind, is a powerful tool for predicting tertiary structures of proteins (34, 35). In recent years it has been shown to be remarkably accurate at protein structure prediction, and more recently algorithms have been developed to also predict limited multimeric structures.

Using AlphaFold version 2.3.1 (AlphaFold2) (34, 36) on the Galaxy Australia web-based platform (https://usegalaxy.org.au/), structure predictions of the SAM domain (residues 191–261) were carried out as a monomer as well as different multimers (trimer, tetramer, pentamer, hexamer and heptamer). The predicted template modelling scores (pTM) of the top ranked model for each predicted structure as well as the average per-residue model confidence scores (pLDDT) on a scale from 1 to 100 for the top ranked structures are listed in Table S1 and each of the predicted structures is shown in Fig. S4, *A*–*E*. The pLDDT score is based on a local distance difference test for assessing predicted protein structures (37).

The results give high model confidence scores for the trimer, tetramer, pentamer, and hexamer as well as high pLDDT scores for each of the top-ranked multimeric structures. In contrast, the heptamer exhibited a very low model confidence score and a significantly lower pLDDT score. Only in the case of the pentamer and the hexamer was an intact cyclic overall structure able to be predicted (Fig. S4, *C* and *D*). The trimer and the tetramer formed partially cyclic structures which were superimposable on a portion of the hexamer (Fig. S4*C*) however they were not able to be superimposed onto a portion of the pentamer. In all predicted structures, the side chains of Lys234, Asp235, and Arg243 are involved in electrostatic contacts as seen in the crystal structure. Additionally, the hydrogen bond interactions between the main chain atoms of Gly241 and Gly218 of a neighboring monomer are maintained in all the predicted structures as also observed in the crystal structure.

Comparisons were made of the crystal structure of the WT SAM domain and the AlphaFold2 predicted structures for the pentamer and the hexamer (Fig. S5, *A* and *B*). This shows a relatively close superposition for the predicted hexamer and the crystal structure (Fig. S5*B*). The positions of all secondary structure elements are conserved in the prediction, relative to the experimental structure. When the central monomer (B) of the three molecules in the asymmetric unit is superimposed on one molecule of the predicted hexamer, the rms displacement of the Cα positions (for the core regions of the domain) of the other two monomers (A and C) are 3.0 Å and 2.7 Å respectively, with chains A and C shifted “upwards” and “downwards” respectively from the corresponding chains in the crystal structure. In the case of the predicted pentamer and the crystal structure, superposition of monomer B results in an rms displacement of the Cα positions (for the core regions of the domain) of 3.5 Å for chain A and 4.6 Å for chain C (Fig. S5*A*). As expected, while the secondary structure elements are conserved in the prediction, the predicted pentamer differs more from the arrangement of three molecules in the crystal structure, due to the shift necessary to place the molecules on the same plane relative to one another, as was required for the predicted hexamer, as well as the need to arrange the molecules into a tighter cyclic pentamer rather than a cyclic hexamer.

Further AlphaFold2 predictions were also carried out for the full-length Tric1, incorporating both the SAM and the PRAT domains (Fig. S6*A*). This showed structural conservation for the experimentally determined SAM domain as well as a possible fold for the PRAT domain. The two domains are connected by an extended linker region which exhibits lower pLDDT scores. The pLDDT score for the Tric1 monomer was overall lower than for the SAM domain alone (Table S1) however the scores for the four predicted transmembrane helices of the PRAT domain (residues 55–79 and 106–127, 137–154 and 162–186) and the residues of the SAM domain (residues 196–261) are considerably higher (>85) than the N-terminal helix (1, 2, 3, 4, 5, 6, 7, 8, 9, 10, 11, 12, 13, 14, 15, 16, 17, 18, 19, 20, 21, 22, 23, 24, 25, 26, 27, 28, 29, 30), the interdomain linker (185–195) and a predicted helical insertion between the first and second TM helices (residues 80–105) (Fig. S6*A*). Thus, the prediction gives a good level of confidence for the core regions of the Tric1 structure, with lower predictions, particularly for the N- and C-termini and the linker regions between the PRAT and SAM domains.

Further predictions were carried out for different multimers of the full-length Tric1 with scores for the predictions shown in Table S1. The results of these predictions showed high pLDDT scores (>80) for the SAM domains in all cases. While the scores for the PRAT domains were considerably lower, as the oligomer increased to a pentamer or a hexamer (Fig. S6, *B* and *C*) the model confidence and pLDDT scores increased, particularly for residues making up the last two transmembrane helices of the PRAT domain (residues 137–154 and 162–186). These predictions showed that a closed oligomer could be formed for the pentamer and hexamer, with the same interchain interactions (involving the side chains of Lys234, Asp235 and Arg243 and the main chain atoms of Gly241 and Gly218), although the exact arrangement of the PRAT domains within the oligomer could not be predicted with high confidence (Fig. S6, *B* and *C*). This may be due to the ten-residue extended linker sequence separating the PRAT and the SAM domains for each monomer. Interestingly, from the predictions obtained, a cyclic pentameric structure results in a pore through the center of the oligomer that is approximately 13 Å wide at the position of the SAM domains (Fig. S7*A*). In the case of the hexameric structure the pore has a width of approximately 20 Å at the position of the SAM domains (Fig. S7*B*). In both cases, the electrostatic nature of the pore shows a positively charged surface throughout, supporting an expected pathway for a negatively charged tRNA molecule to pass through the oligomer, and presumably across the mitochondrial membrane (Fig. S7). Furthermore, the width of an unfolded but still helical tRNA molecule is ∼18 Å supporting the hypothesis that the tRNA is able to thread through the pore of a hexamer.

**Figure 6:**
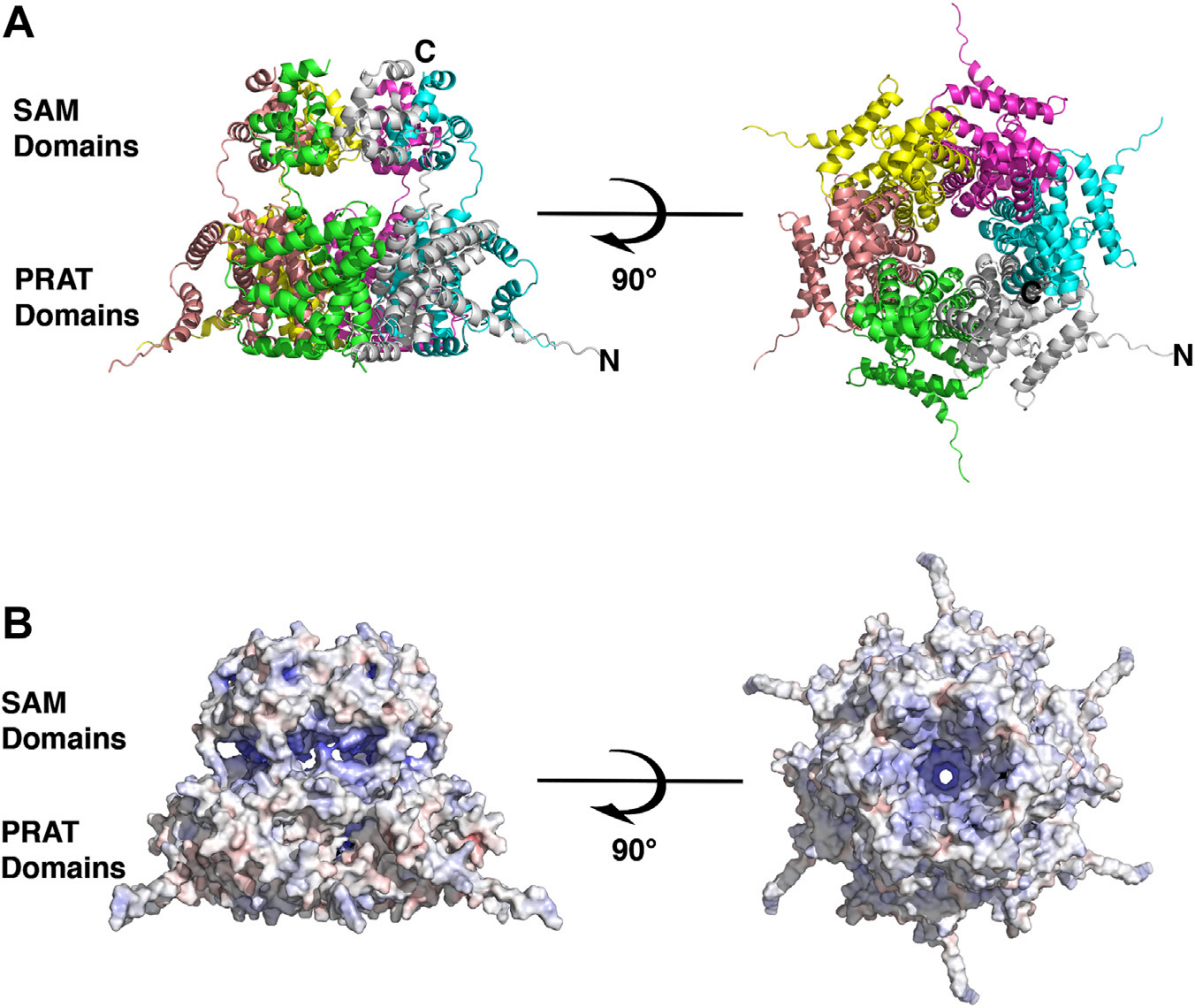
**AlphaFold2 predicted structure of full-length Tric1.***A*, a ribbon representation of the predicted structure of the full-length Tric1 as a hexamer. Each monomer is shown in a different color. The N and C termini have been labeled for a single monomer in each of the orientations shown in the hexameric structure. *B*, an electrostatic surface representation of the hexamer. The *left panels* show the side view of the hexamer and the *right panels* show the *top* view from the side containing the SAM domains. Regions of positive potential are colored in *blue* and regions of negative potential are colored in *red*.

Based on the structural results observed for the SAM domain as well as the AlphaFold2 predictions for both the SAM domain and for the full-length protein, we hypothesize that Tric1 forms a hexameric ring structure (Fig. 6) to facilitate movement of tRNA across the membrane through a pore in the oligomeric assembly.

## Discussion

The mitochondrial outer membrane proteins, Tric1 and Tric2, contain a C-terminal SAM domain that is required for the efficient uptake of tRNA into plant mitochondria (3). Conserved lysine residues within the SAM domain were identified to be essential for tRNA binding ability *in vitro* (3). Inactivation of both Tric1 and Tric2 in *Arabidopsis* (*tric1:tric2*) resulted in severely chlorotic and developmentally delayed plants, defective in tRNA import ability that could not be restored by complementation using the Tric1 protein lacking the SAM domain, suggesting an essential role (3).

To further characterize the structural and functional properties of the Tric1/2 SAM domain, we determined its crystal structure to 1.5 Å resolution. The crystal lattice reveals a helical superstructure of six monomers per helical turn. Furthermore, solution studies indicate an oligomeric species that is highly thermally stable suggesting that the oligomerization behavior of the protein is not a result of crystallization, but rather it is a natural characteristic of the SAM domain. This self-oligomerization is typical of other SAM domain containing proteins, and this feature has been implicated in a wide range of protein functions such as mediation of protein-protein interactions, signaling cascades, transcription and DNA repair activities, often associated with the mode of oligomerization (20, 38, 39, 40, 41, 42).

Mutational and structural studies indicate that the incorporation of a longer side chain at position 241 negatively impacted the hexameric helical structure while mutating Asp235 to an alanine affected the stability of the helical superstructure. EMSA assays, carried out using Asp235Ala and Gly241Glu mutants, reveal, in both cases, abolished RNA binding capability. This suggests that the oligomerization ability of the SAM domain is required for tRNA binding, and that removal of the salt bridge between adjacent SAM domains can also alter RNA binding ability.

To assess the function of both residues *in planta*, complementation assays were carried out. The double mutant variant (Asp235Ala:Gly241Glu) of the full-length Tric1 was tested and failed to restore the aberrant growth phenotype of the double knock-out line *tric1:tric2*, unlike the full-length WT Tric1. This suggests that one or both of these residues (Asp235 and Gly241) are required for the functionality of Tric1.

The protein residues previously indicated to be involved in RNA binding (Lys205 and Lys210) (3) reside on the exterior surface of each monomer in the superhelical structure. An inspection of the electrostatic characteristics of the oligomeric structure shows the outer surface as more positively charged (Fig. S2). Additionally, each hexameric ring exhibits a charge polarity, with the upper surface of the ring (C-terminal end) more negatively charged and the lower surface (N-terminal end) more positively charged. It should be noted however that the final three residues at the C-terminal end of the domain (Lys259, Arg260, Lys261) are not modeled in the structure as there was insufficient electron density. This may suggest that the C-terminal surface is more positively charged than appears in the structure. Thus, although the outer surface residues, Lys205 and Lys210, were shown to be involved in RNA binding, the positively charged electrostatics of the entire surface of the SAM domain including the outer surface may suggest that more residues than only Lys205 and Lys210 may be involved in RNA interaction. Furthermore, the RNA binding studies carried out by Murcha *et al.* (6) as well as the studies reported here, used only the T-arm of tRNA. It may be that other residues are involved in binding different regions of the full tRNA. The N-terminus of the SAM domain lies along the outer and bottom (positively charged) face of the hexameric ring. In the full-length Tric1/2, the PRAT domain, containing four predicted transmembrane alpha helices, would be expected to be positioned near to the N-terminal end of the SAM domain.

SAM domains have been shown to form circular oligomeric structures that are required for protein function (43). For example, in SARM1 (sterile alpha and TIR motif containing 1), a human NAD^+^ hydrolase, an octameric assembly is mediated *via* two tandem SAM domains allowing the formation of an antiparallel double-stranded assembly of a C-terminal TIR (Toll/interleukin-1 receptor) domains (44). The SAM domain containing protein 1 (SAMD1), a repressive chromatin regulator at unmethylated CpG islands, contains a SAM domain that oligomerizes to form a cyclic pentamer (45). The crystal structure of the SAM domain of the polycomb group protein, polyhomeotic, involved in gene repression during development, shows a left-handed helical spiral structure with 6_5_ symmetry similar to what we observe in the Tric1 SAM domain superstructure. It is suggested that this high-order symmetry assists in the organization of higher order chromatin structures. Hence SAM domains are clearly able to adopt different multimeric structures, from closed pentamers, hexamers and octamers to helical superstructures. Guided by these observations, structure predictions were carried out for both the SAM domain as well as the full-length Tric1 as different oligomers. It is apparent from these results that the protein can be arranged as a cyclic pentamer or hexamer.

While, based on the multimeric structure prediction results, a pentameric ring structure cannot be ruled out, we suggest that the protein may be more likely to adopt a hexameric ring structure. The pentameric structure predicted by AlphaFold2 does not generate a pore of the appropriate dimensions to accommodate a double-stranded tRNA molecule (∼18 Å wide). In contrast, the hexameric ring structure does generate a pore of sufficient internal diameter (∼20 Å across the SAM domains portion of the oligomer) through which tRNA is able to pass to cross the mitochondrial membranes. Furthermore, the hexameric oligomer was observed in the crystal structure of the WT and Asp235Ala SAM domains, albeit slightly offset in order to form a helix. In order to validate this hypothesis further structural and biochemical studies need to be carried out with the full-length Tric-1 protein. Specifically, structure elucidation by either CryoEM or crystallography of the full-length Tric1, containing both the SAM domain and the PRAT domain, would provide important experimental insights into the oligomeric state of the protein. Furthermore, structural studies carried out in the presence of tRNA will provide a deeper understanding of the specific molecular features that facilitate RNA interaction and import.

## Experimental procedures

### Clones for protein expression and complementation

Cloning of the full-length Tric1 cDNA (At3g49560) and the Tric1 SAM domain (residues 191–261) is described previously (3). Specifically, the amplified cDNA was cloned into a pET15b vector for protein expression of SAM domain containing an N-terminal hexa-histidine tag and a thrombin cleavage site. The complete sequence of all the constructs can be found in the Table S2. Mutations corresponding to Asp235Ala, Gly241Glu and Asp235Ala:Gly241Glu were introduced into the cloned vectors using the QuickChange II Site-directed mutagenesis kit (Agilent) using primers listed in Table S3.

### Protein expression and purification

The wildtype (WT) Tric1 SAM domain (residues 191–261) and the variants Asp235Ala, Gly241Glu and Asp235Ala:Glu241Gly were recombinantly expressed in *Escherichia coli* Rosetta2 (DE3) using lysogeny broth (LB) containing 50 μg/ml ampicillin and 34 μg/ml chloramphenicol. Protein expression was induced, once an optical density at 600 nm of 0.6 was attained, by the addition of 200 μM Isopropyl β-d-1-thiogalactopyranoside (IPTG) to the growth media and the cells further incubated at 20 °C for 4 h. Cells were harvested by centrifuging at 4000*g* for 45 min at 4 °C, resuspended in buffer A (20 mM HEPES, pH 7.5, 500 mM NaCl, 20 mM imidazole) and flash frozen in liquid nitrogen for storage at −80 °C until further use. The cells were resuspended in buffer A and lysed using an EmulsiFlex-C5 high-pressure homogenizer (Avestin). The cell lysate was centrifuged at 24,000*g* for 45 min and the supernatant applied to 2.5 ml of NiNTA resin (Thermo Scientific) pre-equilibrated with buffer A. The beads were washed with 20 ml of buffer A, followed by 20 ml of buffer B (20 mM HEPES, pH 7.5, 500 mM NaCl, 60 mM imidazole) and the target protein eluted with 10 ml of buffer C (20 mM HEPES, pH 7.5, 500 mM NaCl, 250 mM imidazole). The fractions containing the protein of interest were applied to a Sephacryl 300 16/60 gel filtration column (GE Healthcare) and eluted with buffer D (10 mM HEPES, pH 7.5, 50 mM NaCl). Protein elution was monitored by absorbance at 280 nm. The purified protein was concentrated to a final concentration of 8 to 12 mg/ml, flash frozen in liquid nitrogen and stored in 200 μl aliquots at −80 °C until further use for crystallization experiments and biochemical analyses.

### SeMet protein production and purification

A selenomethionine (SeMet) variant of the Tric1 SAM domain was recombinantly expressed using the methionine inhibition pathway approach (46, 47) in *E. coli* BL21 (DE3). Seven milliliters of inoculant grown overnight from a single transformed colony was added to M9 minimal media (500 ml) supplemented with 1 mM magnesium sulfate, 0.2% (w/v) glucose 10^−5^% thiamine, 0.5 mM calcium chloride, and 50 μg/ml ampicillin. Amino acids were added to the minimal media containing the bacteria to a final concentration of 125 mg/L for lysine, 100 mg/l for phenylalanine and threonine, 50 mg/l for isoleucine, leucine and valine and 60 mg/l for L-selenomethionine. Cells were incubated for 1 h at 37 °C, protein expression was induced by the addition of 0.4 mM IPTG, for 6 h at 20 °C and the cells were then cooled to 4 °C for 30 min. The bacteria were pelleted by centrifugation at 4000*g* for 45 min at 4 °C and lysed as described above. The SeMet variant protein was purified by NiNTA affinity chromatography using Buffers A and C above supplemented with 10 mM MgCl_2_ and 10 mM βME. The eluted protein was dialyzed into 10 mM HEPES pH 7.5, 50 mM NaCl, 1 mM βME and concentrated to 12 mg/ml final concentration for crystallization.

### Analysis of oligomeric formation in solution

To assess the formation of oligomers in solution, 1 ml of purified WT SAM domain and single mutants at 9.5 mg/ml was loaded onto a Superdex 75 16/60 gel filtration column (GE Healthcare) at 1 ml/min pre-equilibrated in buffer D. Samples of the eluted peaks were mixed with 5× Laemmli sample buffer (250 mM Tris-HCl pH 6.8, 4%(w/v) sodium dodecyl sulfate (SDS), 30%(v/v) glycerol, 0.6 g/l bromophenol blue). For WT SAM domain, 3 μl of each sample was heated at 95 °C for 10 min and loaded onto a 15%(w/v) SDS-PAGE gel with a 5.2% stacking gel alongside 3 μl of the same sample that had not been heated, and 5 μl of prestained protein ladder (New England Biolabs). For the single mutants, 10 μg or 16 μl (where the concentration was insufficient to load 10 μg) was heated and loaded onto a gel as described for the WT. The gels were electrophoresed at a constant 35 mA until the dye front was 0.5 cm from the base of the gel and stained with Coomassie. In the case of WT SAM domain, a second gel was then blotted onto a nitrocellulose membrane (GE Healthcare) using the XCell II blot module (Thermo Fisher) at a constant current of 170 mA for 1 h at 4 °C. The membrane was incubated in 20 ml blocking buffer (50 mM Tris-HCl pH 7.5, 150 mM NaCl, 0.05% (v/v) TWEEN 20, 5% (w/v) skim milk powder) for 1 h at RT with gentle shaking. A monoclonal anti-polyhistidine-peroxidase antibody was added at a 1/2000 dilution (Sigma-Aldrich). The membrane was incubated with the antibody for 10 min at RT with gentle shaking. The antibody solution was removed and the membrane was washed 5× with tris-buffered saline with tween (TBST) (50 mM Tris-HCl pH 7.5, 150 mM NaCl, 0.05% (v/v) TWEEN 20) for 10 min each with gentle shaking at RT. The membrane was removed from the TBST and 1 ml of WesternSure chemiluminescent substrate (LI-COR) was pipetted over the surface. The blot was imaged using a chemidoc imaging system (BioRad).

### Crystallization

Crystallization of the Tric1 SAM domain (WT) and variants was carried out by vapor diffusion using the hanging-drop method at 20 °C. Crystallization drops contained 2 μl of the protein solution and 2 μl of the reservoir solution. Rod-shaped crystals of the WT and Asp235Ala were obtained using a protein concentration of 10 mg/ml and a precipitant solution of 1.7 M ammonium sulfate, 0.1 M Tris, pH 8.2. Microcrystals of the Gly241Glu mutant were obtained using protein at 16 mg/ml and a precipitant solution of 2.3 M ammonium sulfate, 0.1 M Tris, pH 8.2. Crystals of suitable size for diffraction experiments were obtained through an iterative microseeding procedure where microcrystals were removed directly from the original drop with a cryo-loop and added to a freshly set up crystallization drop, containing 2.0 M ammonium sulfate, 0.1 M Tris, pH 8.7. Crystals formed in the seeded crystallization drop after gradually increasing the precipitant concentration in the well from 2.0 M ammonium sulfate to a final concentration of 2.3 M. This was done by transferring the coverslip containing the drop to a fresh reservoir solution containing 0.1 M higher precipitant concentration and the drop equilibrated for 1 week. This transfer procedure was repeated a further two times until the crystallization drop was equilibrated against a reservoir solution of 2.3 M ammonium sulfate. The final diffraction quality crystals for the Gly241Glu mutant were obtained from drops equilibrated against 2.3 M ammonium sulfate, 0.1 M Tris, pH 8.7. Crystals of the SeMet mutant protein were obtained by the sitting-drop vapor diffusion method at 20 °C using protein at 6 mg/ml and a precipitant solution of 1.0 M ammonium sulfate, 0.1 M Tris, pH 8.2. Crystals were harvested from the drops using cryo-loops (Hampton Research), cryoprotected in the reservoir condition containing 15% glycerol and flash frozen in liquid nitrogen in preparation for X-ray data collection.

### X-ray data collection, data processing, and structure solution

X-ray diffraction data were collected at the Australian Synchrotron beamlines MX1 and MX2 equipped with an EIGER 9 M detector and an EIGER 16 M pixel detector respectively with continuous readout (‘shutterless’ data collection). All data was collected at a temperature of 100 K from 360° of crystal rotation using the Blu-Ice data acquisition software (48), integrated using the XDS processing package (49) and data reduction carried out using the CCP4 software package (50). Phasing of the SeMet mutant was carried out using the multi-anomalous diffraction method from selenium atoms in the mutant protein. Data from four wavelengths were collected (Table 1) and the selenium positions were determined using the HKL2MAP interface in CCP4 to access the SHELX programs for phasing. Six selenium atoms were located in the asymmetric unit, corresponding to two methionine residues per protein chain (Met202 and Met245). The estimated mean Figure of Merit and the Pseudo-free correlation coefficient obtained from after phasing and density modification with SHELXE was 0.67 and 0.72 respectively. The complete model was built into the electron density map. Refinement was carried out using the SeMet mutant model structure against the high-resolution WT data (Table 1), replacing the selenium atoms in the model for sulfur atoms. The structures of the Asp235Ala and Gly241Glu variant proteins were solved by molecular replacement with the program PHASER (50) using the WT Tric1 SAM domain as the search model. Iterative cycles of model building using the graphic software COOT (51) and crystallographic refinement using the PHENIX software suite (52) was carried out. The data reduction and refinement statistics are given in Table 1. All structures were visualized using Open-Source PyMOL (http://www.pymol.org/pymol).

### Protein structure prediction

Artificial intelligence-guided 3D protein structure predictions were carried out using AlphaFold version 2.3.1 (AlphaFold2) (34, 36) on the Galaxy Australia web-based platform (https://usegalaxy.org.au/). The parameters used for the configuration of AlphaFold2 were as described on AlphaFold’s Github site (https://github.com/google-deepmind/alphafold/tree/main). Specifically, the AlphaFold-Multimer weights were based on a PDB training cutoff of 2021-09-30. Predictions were carried out on the Tric1 SAM domain alone (residues 191–261) and the full length Tric1 as monomers. Predictions of multimers were carried out using AlphaFold2 in ‘Multimer” mode where multiple sequences (depending on the oligomeric state being predicted) were input in FASTA format. All predictions were carried out using the full database for enhanced accuracy.

### RNA electromobility shift assays using fluorescein-labeled RNA oligonucleotides

RNA electromobility shift assays were performed using 5′-(6-FAM)-labeled RNA oligonucleotides corresponding to the T-arm of tRNA^ala^ (Table S3). Fluorescein-labeled tRNA^ala^ oligonucleotides (120 nM) were incubated for 15 min at 22 °C in a final volume of 20 μl containing 160 μM purified protein in a binding buffer (15 mM Tris-HCl pH 7.0, 150 mM NaCl, 10% (w/v) glycerol) and 2 units/μl RNase inhibitor RiboShield (PCR Biosystems). Following the incubation 2 μl of DNA loading dye (New England Biolabs) was added to the reaction mixture, bringing the final volume to 22 μl, in order to provide a visible dye front. The reaction was resolved on a nondenaturing 6% Tris/borate/EDTA polyacrylamide gel, in 0.5× Tris/borate/EDTA buffer gel run at 100 V for 45 min at room temperature and imaged using an Amersham Typhoon scanner (GE Healthcare).

### Plant growth conditions

*A. thaliana* cv Columbia (Col-0), *tric1:tric2* and complementation lines WT line 1 & 2 and Asp235Ala:Gly241Glu lines 1 & 2 were grown plants on soil-vermiculite-perlite mix (3:1:1) under standard long-day conditions (22 °C, 16 h day and 8 h night, ∼100 μmol m^−2^ s^−1^ light intensity). Plants were imaged and rosette leaves were harvested at 8 to 10 weeks post sowing.

### Complementation of *tric1:tric2*

Complementation of the *tric1:tric2* knockout (3) was carried out using *Agrobacterium tumefaciens*-mediated floral dipping transformation (53). Two independent T_3_ complementation lines were used for all analyses. Chlorophyll content estimation was determined using the SPAD-502 chlorophyll meter (Minolta) and averaged from five technical replicates across one leaf from 10 individual plants (*n* = 10). Significant differences were determined using one way ANOVA from the Brown-Forsythe and Welsch tests with the statistical analysis program GraphPad Prism (version 9.0.0).

### Figure generation

Adobe Illustrator, Adobe Photoshop, GraphPad Prism, and PyMol were used for figure generation and structure visualization.

## Data availability

The X-ray crystal structure data presented in this study have been deposited to the Worldwide Protein Data Bank. The PDB accession codes for the WT, Asp235Ala and Gly241Glu Tric1 SAM domains are 8UCY, 8UCZ and 8UD0 respectively.

## Supporting information

This article contains supporting information.

## Conflict of interest

The authors declare that they have no known competing financial interests or personal relationships that could have appeared to influence the work reported in this paper.
