# Supplementary material for: Structural analysis of the SAM domain of the Arabidopsis mitochondrial tRNA import receptor
Source: J Biol Chem. 2024 Apr 4;300(5):107258. doi: 10.1016/j.jbc.2024.107258 (PMC11063897; doi:10.1016/j.jbc.2024.107258)
Supplement: Supporting Figures S1–S7 and Tables S1–S3 [file mmc1.docx]

**Supplementary Figures and Tables**


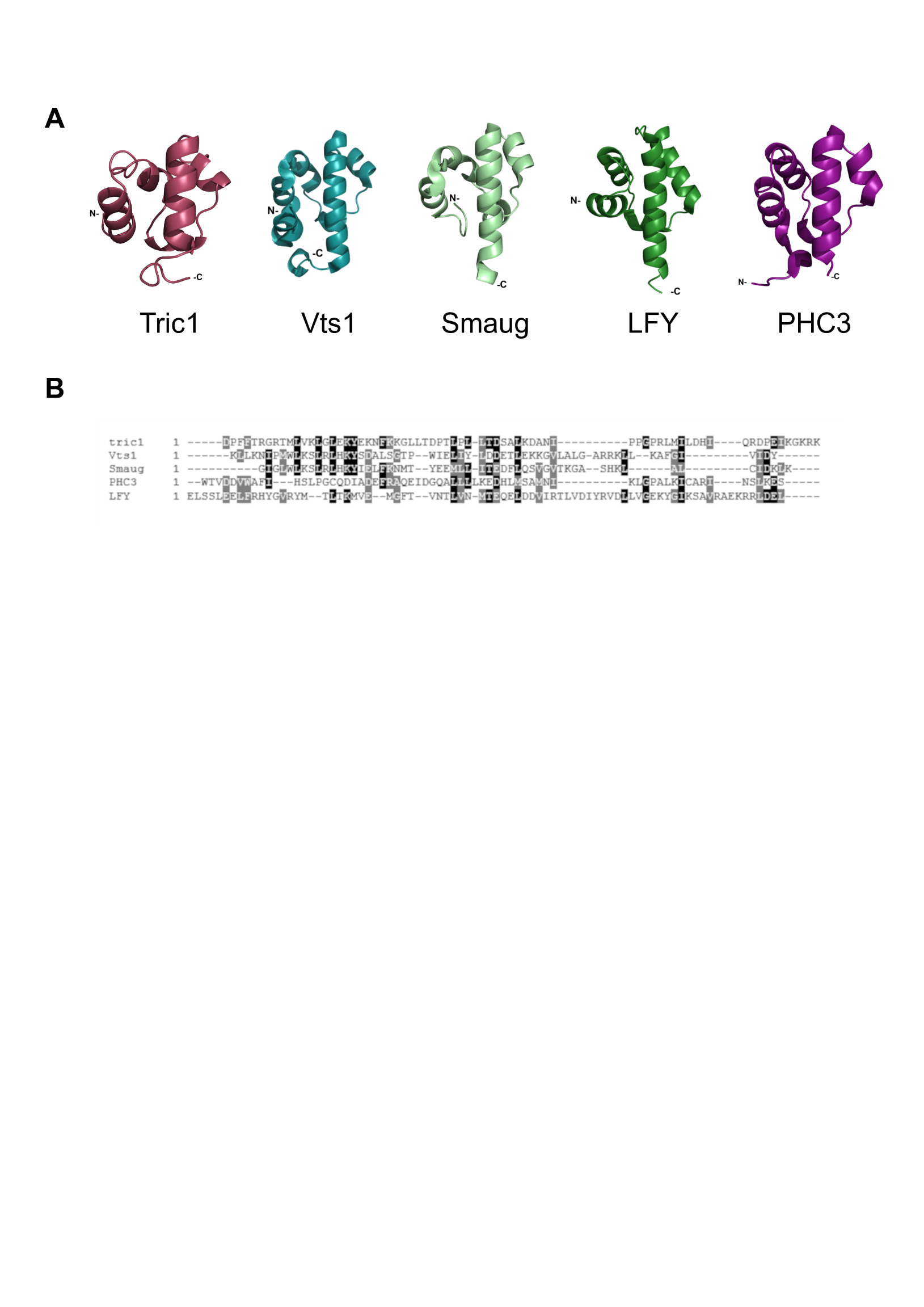


**Supplementary Figure 1: Comparisons of SAM domain structure from different proteins. A.** Tric1: plant RNA binding protein involved in the transport of tRNA into the mitochondria. Vts1: yeast RNA binding protein involved in post transcriptional regulation (PDB code 2FE9). Smaug: Drosophila melanogaster RNA binding protein required for abdominal segmentation in early embryos (PDB code 1OXJ). LFY: plant SAM domain containing protein involved in DNA binding regulating floral transition (PDB code: 4UDE). PHC3: human protein required to maintain the transcriptionally repressive state of many genes throughout development (PDB code 4PZO). **B.** Sequence alignment of the SAM domains from each of the protein displayed in panel A).


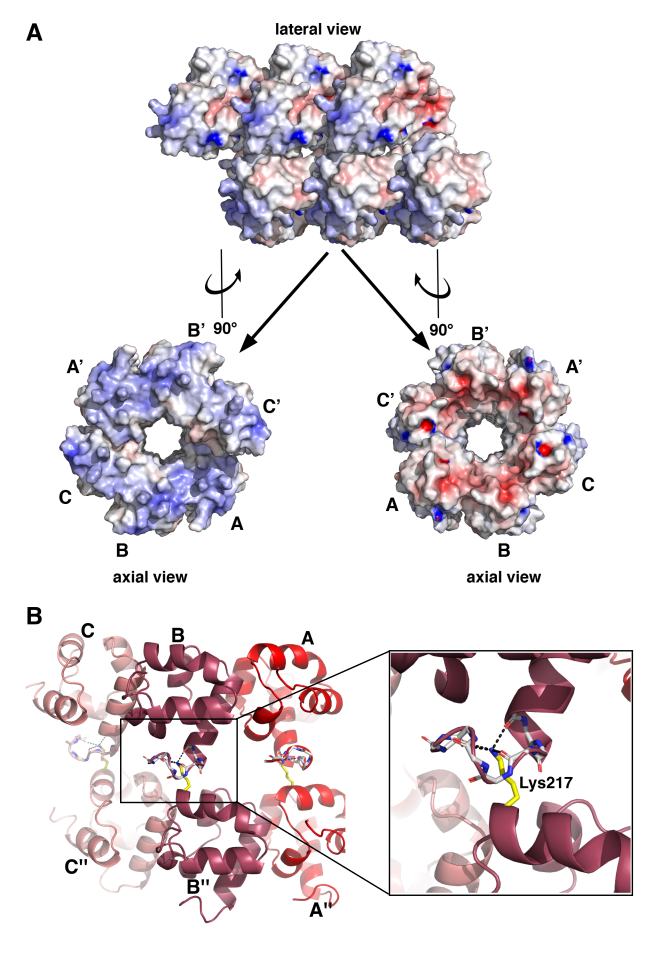


**Supplementary Figure 2:** **Helical superstructure of the SAM domain.** **A.** An electrostatic surface representation. The top panel shows a lateral view consisting of 18 monomers of the SAM domain. The bottom two panels show the two axial views corresponding to 90° rotations of the helical superstructure. The monomers in a single asymmetric unit of the crystal structure are indicated by A, B and C and those in a second asymmetric unit are labelled as A’, B’ and C’. Regions of positive potential are colored in blue and regions of negative potential are colored in red. **B.** The intermolecular interactions between the monomers along the helical axis. The monomers A, B and C correspond to one asymmetric unit. The monomers A”, B” and C” correspond to a second asymmetric unit parallel to the helical axis. The inset shows the intermolecular hydrogen bond contacts (shown in dotted lines) between Lys217 of molecule B” and the main chain oxygen atoms of residues 79, 82 and 85 of molecule B. The side chains of these latter residues have been removed for clarity.

**
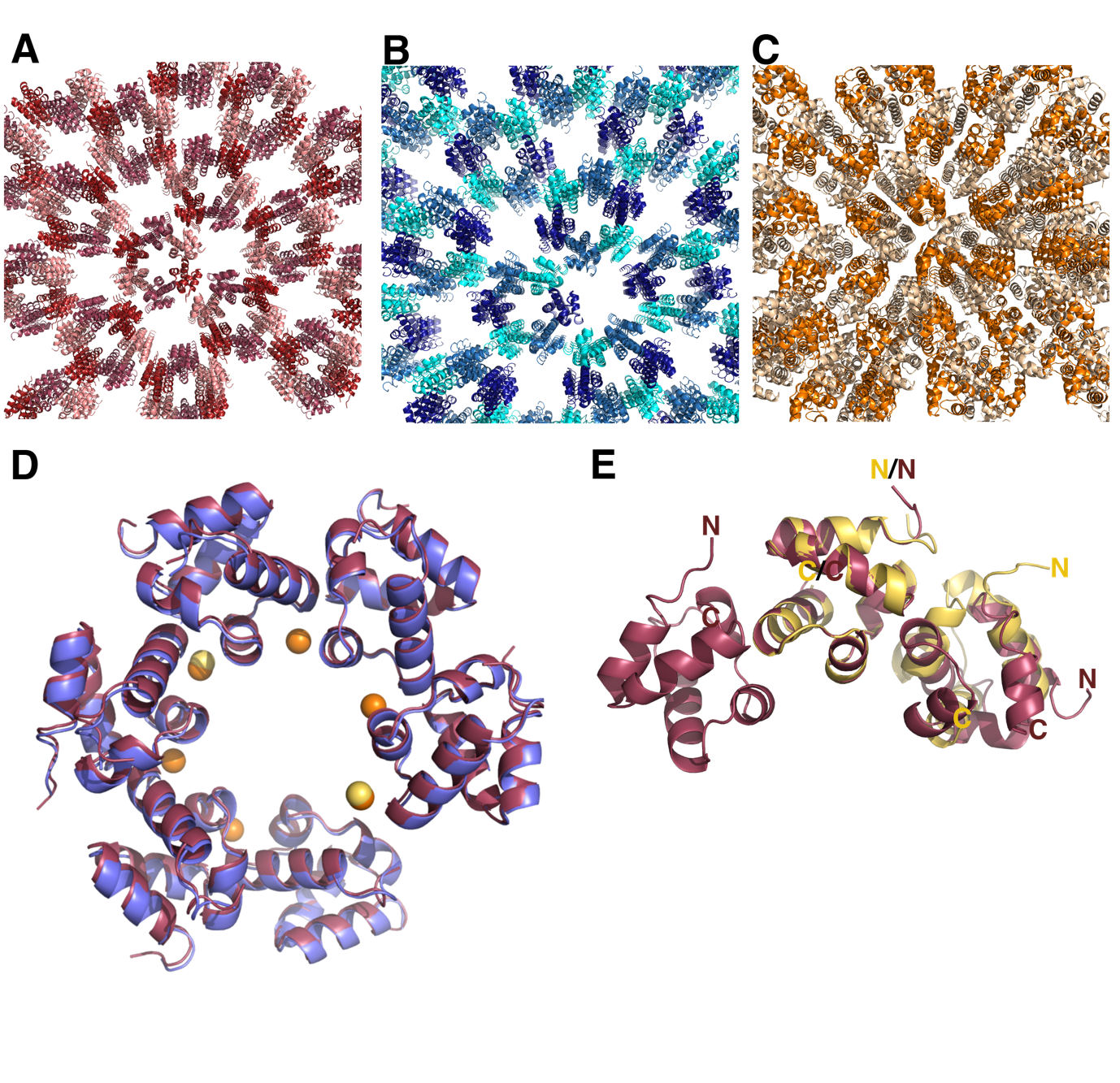
Supplementary Figure 3: Crystallographic lattice and structure comparisons of the WT Tric1 Sam domain and mutant structures. A.** The crystal lattice of the WT protein **B.** The crystal lattice of the Asp235Ala mutant **C.** The crystal lattice of the Gly241Glu mutant. The monomers in the asymmetric units are colored as shown in Figures 1 and 2. **D.** Superposition of the structures of WT Tric1 SAM domain and the Asp235Ala mutant. Two asymmetric units are shown corresponding to 6 molecules. The red colored molecule corresponds to the WT structure and the purple colored molecule corresponds to the Asp235Ala structure. The modelled chloride ions are also included in the superposition as 2 yellow and 6 orange spheres (for the WT and Asp235Ala structures respectively). **E.** Superposition of the structures of WT Tric1 SAM domain and the Gly241Glu mutant. One asymmetric unit is shown corresponding to 3 molecules for the WT structure and 2 molecules for the Gly241Glu mutant structure. The red coloured molecule corresponds to the WT structure and the yellow colored molecule corresponds to the Gly241Glu structure. The N and C termini for each chain are labelled in the color corresponding to the chain.


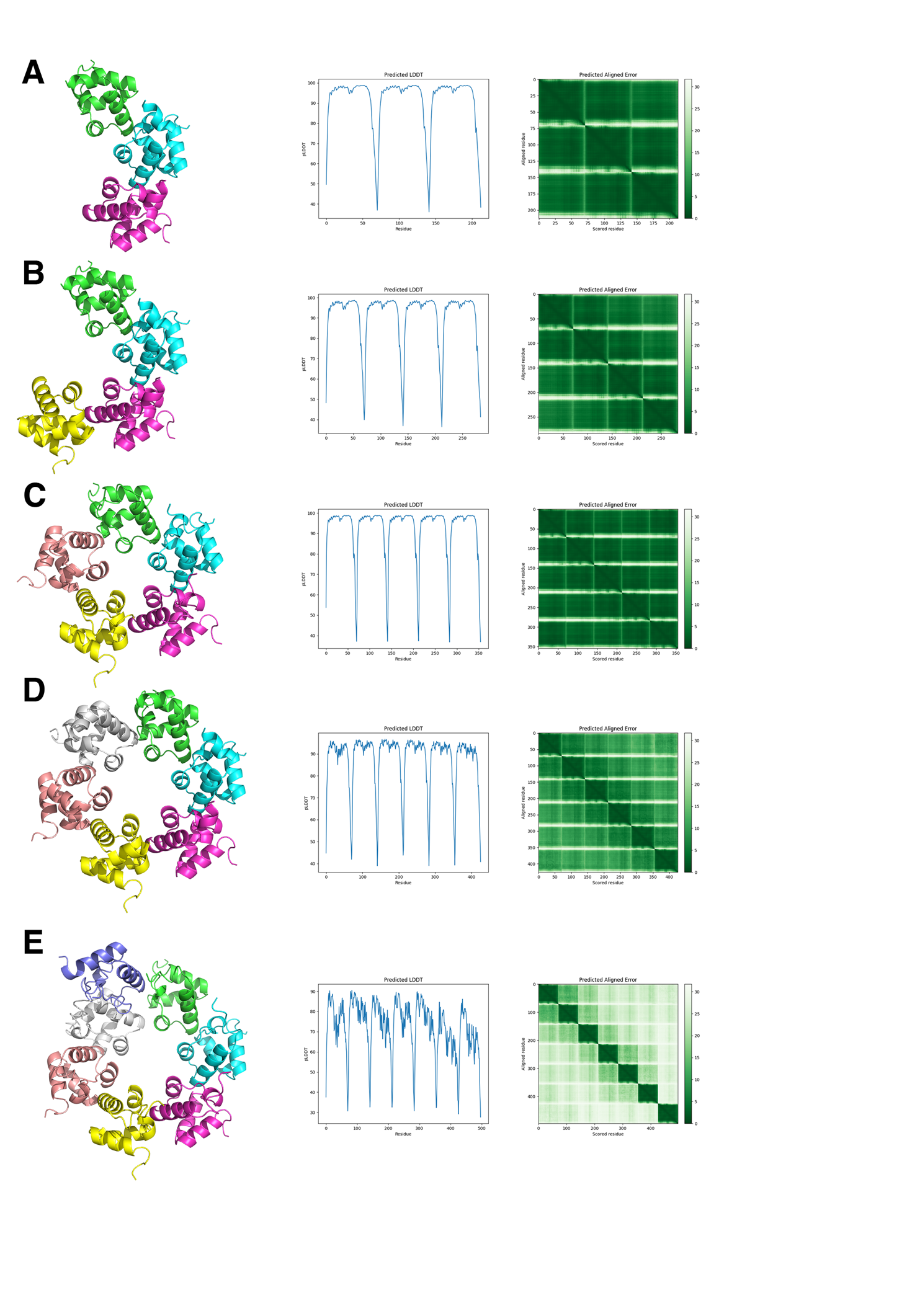


**Supplementary Figure 4:** **AlphaFold2 predicted structures of the Tric1 SAM domain (residues 191-261).** A ribbon representation of the predicted structures as **A.** a trimer, **B.** a tetramer, **C.** a pentamer, **D.** a hexamer and **E.** a heptamer. The pLDDT scores and the predicted alignment error analyses as a function of residues as output from AlphaFold2 are shown to the right of each predicted structure. Each monomer in the multimeric structures are shown in a different color.


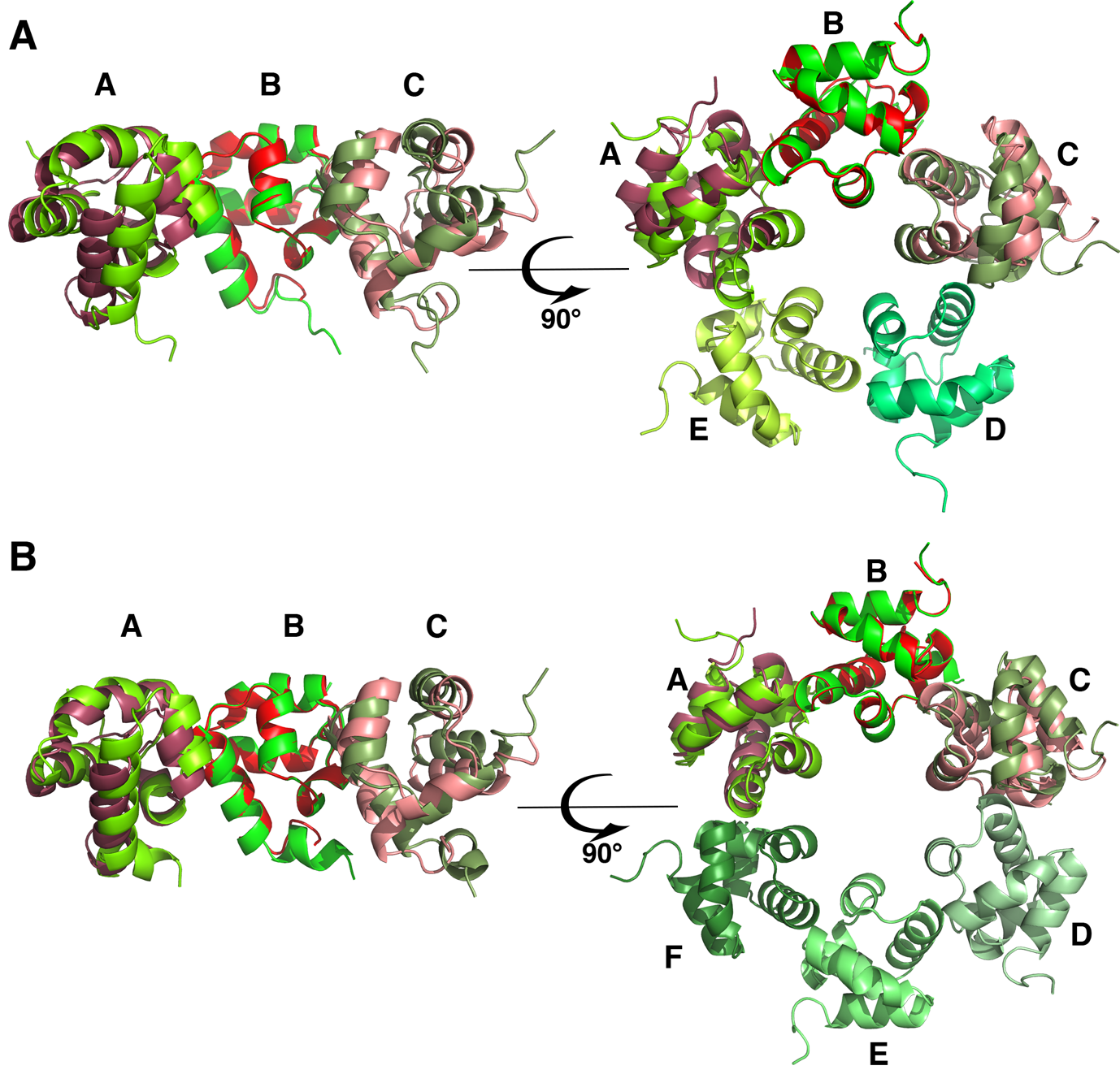


**Supplementary Figure 5. Comparisons of the AlphaFold2 predicted structures and the crystals structure of SAM domain.** **A.** Ribbon representations showing the superposition of 3 molecules from the asymmetric unit of the crystal structure (colored in different shades of red) with the predicted pentamer (colored in different shades of green). The left panel shows only 3 molecules of the predicted pentamer while the right panel (rotated by 90 °) shows all 5 molecules in the pentamer. **B.** Ribbon representation showing the superposition of 3 molecules from the asymmetric unit of the crystals structure (colored in different shades of red) with the predicted hexamer (colored in different shades of green. The left panel shows only 3 molecules of the predicted hexamer while the right panel (rotated by 90 °) shows all 6 molecules in the pentamer.


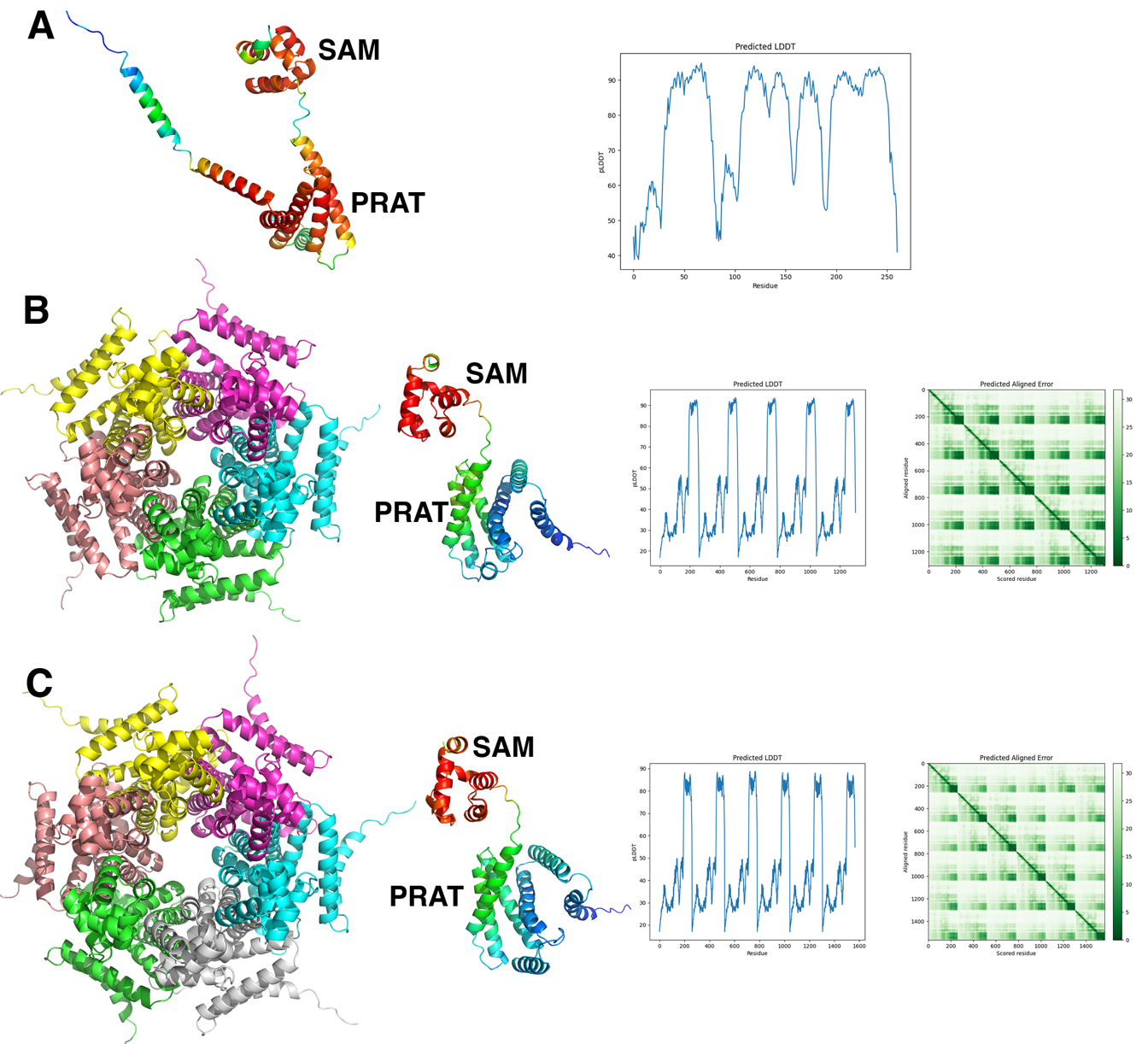


**Supplementary Figure 6:** **AlphaFold2 predicted structures of full length Tric1.** A ribbon representation of the predicted structures as **A.** a monomer, **B.** a pentamer and **C.** a hexamer. For panel A the ribbon representations is colored by pLDDT score (with increasing score from blue to red). For panels B and C the left ribbon diagram shows the multimer colored by chain and the middle panel shows the monomer, colored by pLDDT score (with increasing score from blue to red). The right panels show the pLDDT scores and the predicted alignment error analyses as a function of residues as output from AlphaFold2. The SAM and PRAT domains are labelled in each panel.


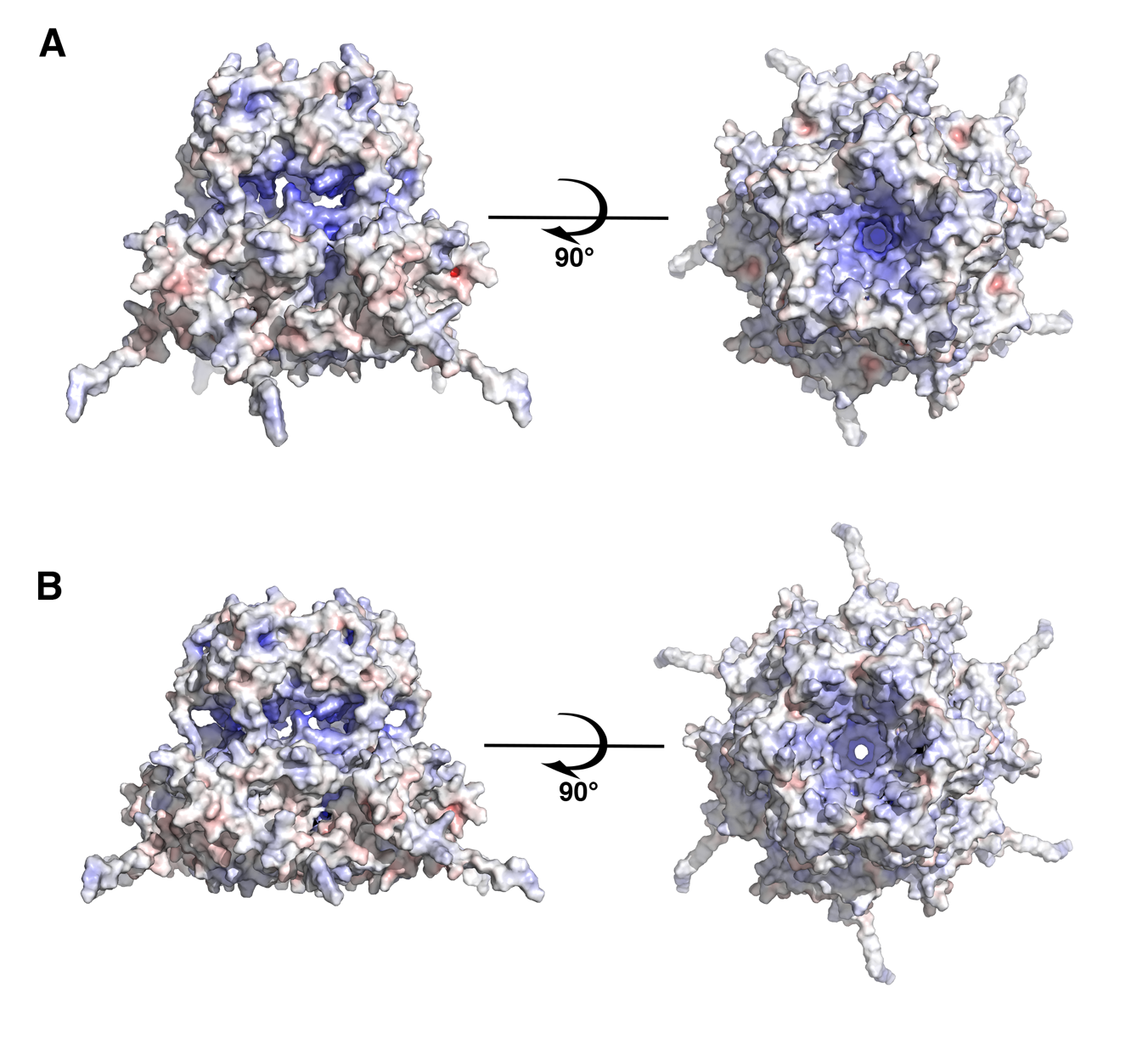


**Supplementary Figure 7: Electrostatic surfaces of full length Tric1 pentamer and hexamer.** A surface representation of the AlphaFold2 predicted oligomers of **A.** a pentamer and **B.** a hexamer. Each panel is shown in 2 different views. Regions of positive potential are colored in blue and regions of negative potential are colored in red.

**Supplementary Table 1: Structure prediction scores obtained from AlphaFold2 calculations on SAM domain and on full length Tric1**

| **Multimer** | **Tric1 SAM Domain**  **(pTM Score/Average residue pLDDT score for top ranked structure)** | **Full length Tric1**  **(pTM Score/Average residue pLDDT score for top ranked structure)** |
| --- | --- | --- |
| Monomer | 91.87/91.4 | 78.29/78.7 |
| Trimer | 0.86/91.0 | 0.33/45.8 |
| Tetramer | 0.84/90.6 | 0.27/38.7 |
| Pentamer | 0.90/91.8 | 0.52/50.5 |
| Hexamer | 0.71/87.5 | 0.43/48.4 |
| Heptamer | 0.29/72.4 | Not determined |

**Supplementary Table 2: Amino acid sequence of the SAM domain expression constructs**

| **Construct** | **Sequence** |
| --- | --- |
| **WT** | **MGSSHHHHHHSSGLVPRGSTEDPFFTRGRTMLVKLGLEKYEKNFKKGLLTDPTLPLLTDSALKDANIPPGPRLMILDHIQRDPEIKGKRK*** |
| **D235A** | **MGSSHHHHHHSSGLVPRGSTEDPFFTRGRTMLVKLGLEKYEKNFKKGLLTDPTLPLLTDSALKAANIPPGPRLMILDHIQRDPEIKGKRK*** |
| **G241E** | **MGSSHHHHHHSSGLVPRGSTEDPFFTRGRTMLVKLGLEKYEKNFKKGLLTDPTLPLLTDSALKDANIPPEPRLMILDHIQRDPEIKGKRK*** |
| **D235A:G241E** | **MGSSHHHHHHSSGLVPRGSTEDPFFTRGRTMLVKLGLEKYEKNFKKGLLTDPTLPLLTDSALKAANIPPEPRLMILDHIQRDPEIKGKRK*** |

**Supplementary Table 3: Primers and RNA sequences**

| Tric1 | SDM primer for Tric1 Asp235Ala | F: TAGCGCGCTGAAAGCTGCGAACATCCC |
| --- | --- | --- |
|  |  | R: GGGATGTTCGCAGCTTTCAGCGCGCTA |
| Tric1 | SDM primer for Tric1 Gly241Glu | F: GAACATCCCACCAGAGCCAAGACTTATGATAC |
|  |  | R: GTATCATAAGTCTTGGCTCTGGTGGGATGTTC |
| tRNA^ala^-T arm | RNA binding | 5’-(6FAM)-UCGCUUUGCAUGCGA |
